# Characterization of the complete mitochondrial genome of *Omosita colon* (Coleoptera: Nitidulidae)

**DOI:** 10.1080/23802359.2021.1915201

**Published:** 2021-04-26

**Authors:** Wang Xu, Yu Wang, Man Wang, Yinghui Wang, Yanan Zhang, Jiangfeng Wang

**Affiliations:** Department of Forensic Medicine, Soochow University, Suzhou, PR China

**Keywords:** Mitogenome, Nitidulidae, evolution, storage pest, necrophagous beetle

## Abstract

*Omosita colon* (Linnaeus, 1758) (Coleoptera: Nitidulidae) is an economically important storage pest worldwide and a forensically important beetle. The first complete mitochondrial genome (mitogenome) of *O. colon* was sequenced in this study using the next-generation sequencing. The mitogenome of *O. colon* is circular with a total length of 16,544 bp, which consists of 13 protein-coding genes, 22 transfer RNA genes, two ribosomal RNA genes, and a non-coding control region. The order and orientation of genes were identical with that of the ancestral insects. This study provides genomic data for mitogenome library of the genus *Omosita* to investigate evolutionary and systematic studies. It also provides a molecular basis to infer the postmortem interval (PMI_min_) with *O. colon*.

## 1. Introduction

**Figure 1.**
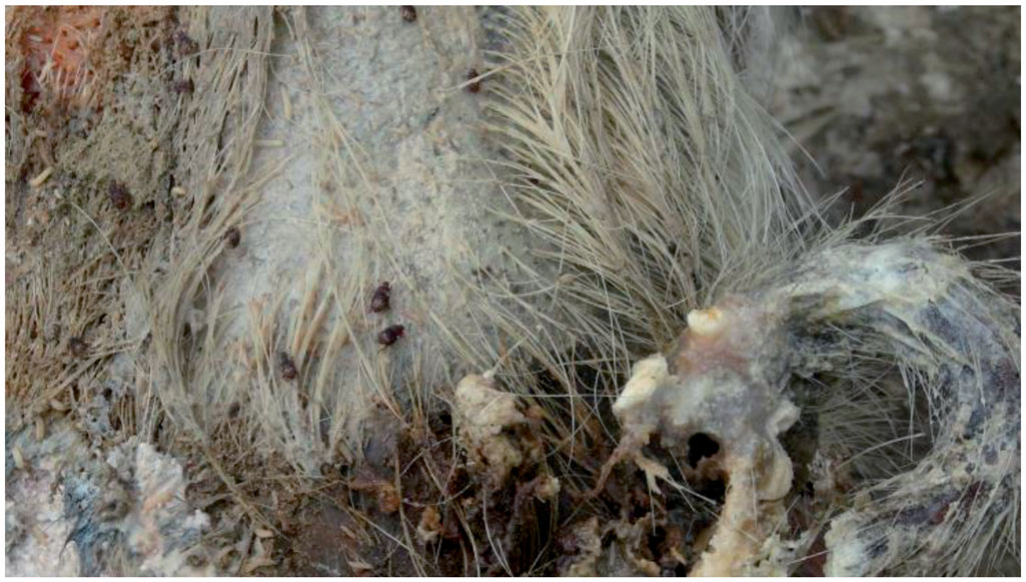
*Omosita colon* on a highly decomposed pig carcass in the field (Suzhou, China).

The genus *Omosita* (Coleoptera: Nitidulidae: Nitidulinae) is a group of Nitidulidae, which is widely distributed in the Holarctic regions (Lee et al. 2015). Some species are considered as important stored-product pests. In this family, *Omosita colon* (Linnaeus, 1758) (Coleoptera: Nitidulidae) is not only a pest of stored Chinese herbal medicines and stored grain products but also a forensically important necrophagous beetle. *O. colon* is closely associated with corpses (Figure 1) and has been reported in many insect fauna succession studies (De Jong and Hoback 2006; Anton et al. 2011; Matuszewski et al. 2013; Lyu et al. 2016). In China, it is widely distributed in most of the provinces and districts (Zhang et al. 2008).

Being an important insect, there have been very few studies on *O. colon.* Most of them focused only on the taxonomy of *O. colon* (Cao and Huang 2016; Jelinek and Audisio 2009). Beyond that, few other aspects of *O. colon* have also been reported. For example, Wang et al. (2020) studied the development of *O. colon* at seven constant temperatures between 16 and 34 °C, which provided fundamental development data that supported use of *O. colon* in minimum postmortem interval (PMI_min_) estimations. Molecular information on *O. colon* is inadequate, and only a few reports mentioned about the taxonomic study of beetle species in certain areas (Pentinsaari et al. 2014; Hendrich et al. 2015). On commencement of the study, there were only 13 partial gene sequences of *O. colon* in GenBank, and the characterization of the mitochondrial genome (mitogenome) of *O. colon* has not been conducted till date. However, the molecular data of *O. colon* is considered significant as it provides the basis for the taxonomic study of beetles. On the other hand, it establishes the molecular basis for PMI_min_ estimation with *O. colon* in the field of forensic medicine. Therefore, it is not only of scientific significance but also of practical application value to study the mitogenome of *O. colon*.

Mitogenome is a common molecular marker for evolutionary, phylogenetic, and population genetic studies, which provides information for analyses of several taxonomic levels (Drosopoulou et al. 2019). Besides, when molecular identification of a species is required, it is helpful to select the most suitable mitochondrial marker by analyzing the complete mitogenome. The structure of insect mitogenome is relatively conservative consisting of 37 genes: 13 protein-coding genes (PCGs), 22 transfer RNA (*tRNA*) genes, and two ribosomal RNA (*rRNA*) genes (Song et al. 2016; Feng et al. 2018). Recently, the mitogenome has been widely used in the phylogenetic studies (Burger et al. 2003; Charles 2005). The genome organization, gene arrangement, preference of the codons, and tRNA structure in the mitogenome can be used in the phylogenetic tree construction (Qiang et al. 2018; Wang et al. 2018). So far, no studies have been conducted on mitogenome of the genus *Omosita*. In this study, the complete mitogenome of *O. colon* was sequenced and assembled by next-generation sequencing. In conclusion, we report the first complete mitogenome of *O. colon*, along with analysis of its gene arrangement, and phylogenetic analysis of nitidulid beetles. This study provides a complete genomic data for mitogenome library of the genus *Omosita* to investigate both evolutionary and systematic studies, and to provide a molecular basis to infer the PMI_min_ with *O. colon.*

## 2. Materials and methods

### 2.1. Insects

The *O. colon* specimens used in this study were collected from a pig carcass placed in a field environment in Suzhou, China (31°21′N, 120°53′E) in April 2018. Species identification was conducted under a Zeiss 2000-C stereomicroscope (Jena, Germany) according to the identification keys provided by Zhang et al. (2008). The captured adults were reared under a constant temperature incubator for further study.

### 2.2. DNA extraction, sequencing, and assembly

Total genomic DNA was extracted from single *O. colon* using Rapid Animal Genomic DNA Isolation Kit (Sangon Biotech, Shanghai, China) following the manufacturer’s instructions for total DNA purification from animal tissues. 1% agarose gel electrophoresis (voltage: 200 V, time: 30 min) was used to detect DNA integrity, and Qubit was used to detect the concentration of DNA samples.

On obtaining the total DNA of *O. colon*, we used DNA Library Prep Kit from Illumina (NEB, Ipswich, MA) for library preparation. Sequencing was constructed on Illumina Hiseq2500 Platform with HiSeq PE150 mode (Paired-end) (Sangon Biotech, Shanghai, China).

Prior to quality control, the statistical information of BBtools was used to evaluate the quality of the original data, and some basic information was counted and visualized to determine the data quality. The raw data was then quality-controlled using BBduk and BLAST+. After that, we used the NOVOPlasty software for *de novo* assembly of the mitogenome of *O. colon* with the obtained clean reads. If the result was not desirable, we extracted the mapped reads, used Spades version 3.13.0 (St Petersburg, Russia) for splicing, and tried to choose the appropriate contig connection mode through Blast+.

### 2.3. Annotation and analysis

The genes of the mitogenome were predicted using MITOS2 Server (http://mitos.bioinf.uni-leipzig.de/index.py) (Bernt et al. 2013). Then, *tRNA* genes were predicted using tRNAscan-SE version 1.3.1 (California, USA) (Lowe and Chan 2016) and ARWEN (Laslett and Canb 2008). Meanwhile, MEGA version 7.0 (Auckland, New Zealand) (Kumar et al. 2016) was used to analyze the nucleotide composition, and relative synonymous codon usage (RSCU) was analyzed by Biopython. Strand asymmetry of the mitogenome was assessed using the following formulas: AT skew = [A − T]/[A + T], and GC skew = [G − C]/[G + C] (Junqueira et al. 2004). Finally, the circular map of the complete mitogenome was drawn with OGDRAW (Lohse et al. 2013).

We used data from the newly sequenced mitogenome of *O. colon* and those of 14 other taxa for phylogenetic analysis of the infraorder Cucujiformia (Coleoptera and Polyphaga). As outgroups, we used one species from the infraorder Scirtiformia (Table 1). The bootstrap consensus tree was inferred by the maximum-likelihood (ML) method based on the Kimura 2-parameter model with 1000 bootstrap replicates. All the above alignments, analyses, model selection, and phylogeny reconstruction were performed in MEGA version 10.0 (Auckland, New Zealand).

**Table 1.** GenBank accession numbers of published infraorder Cucujiformia members and outgroup.

Infraorder	Family	Species	Accession number
Cucujiformia	Nitidulidae	*Carpophilus dimidiatus*	MN604384
		*Carpophilus pilosellus*	MN604383
		*Aethina tumida*	MF943248
		*Epuraea guttata*	KX087289
	Cerambycidae	*Anastrangalia sequensi*	KY773687
		*Cortodera humeralis*	KX087264
		*Monochamus alternatus*	KJ809086
	Meloidae	*Hycleus phaleratus*	MF491389
		*Hycleus marcipoli*	KX161857
		*Hycleus cichorii*	MF491388
		*Epicauta ruficeps*	MN913338
	Tenebrionidae	*Blaps rhynchoptera*	MN267802
		*Tribolium audax*	KJ752724
		*Tribolium castaneum*	KM009121
Scirtiformia	Eucinetidae	*Eucinetus haemorrhoidalis*	KX035155

## 3. Results

### 3.1. Mitogenome organization and nucleotide composition

**Figure 2.**
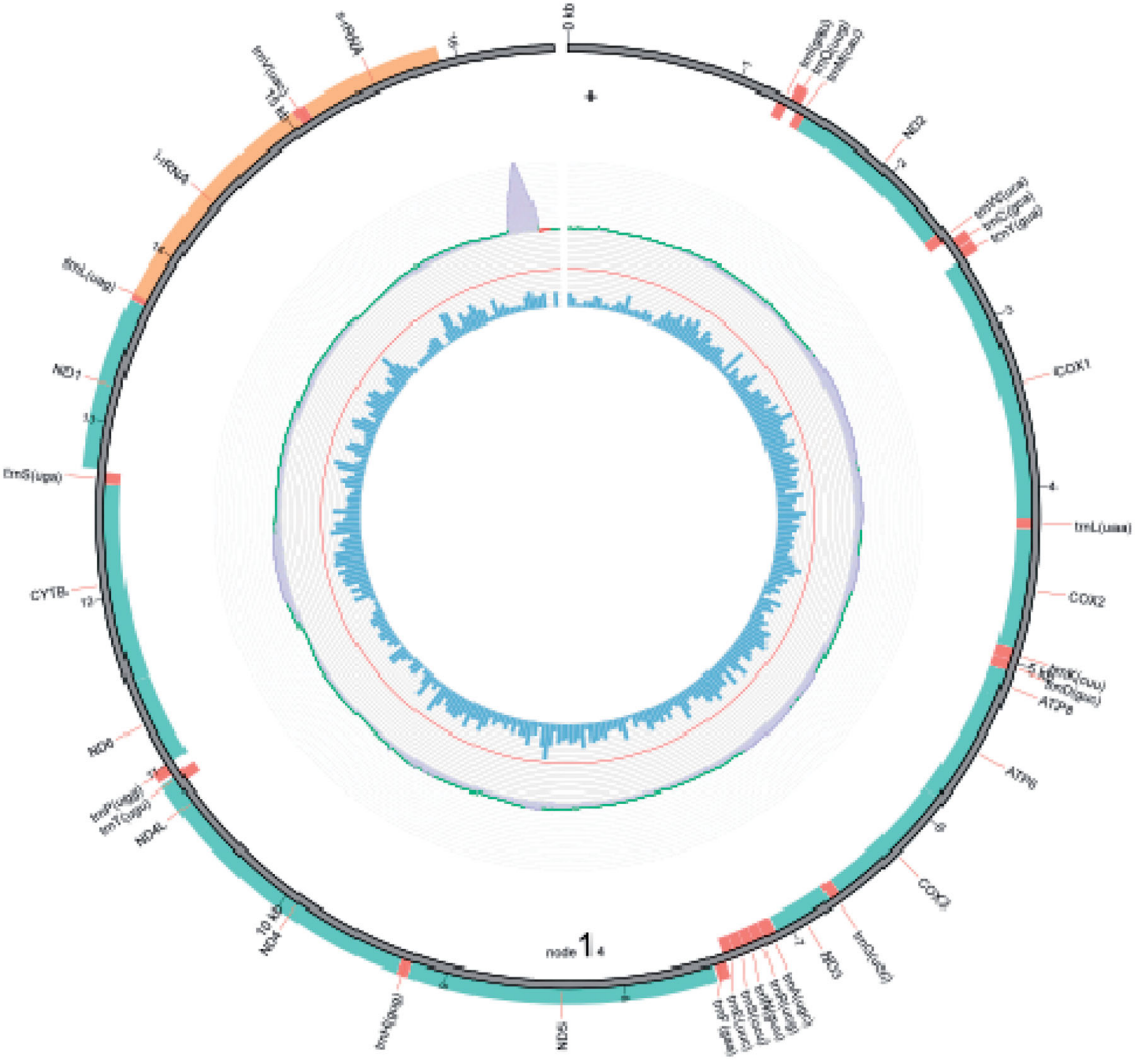
The mitogenome arrangement of *O. colon*. The red color is transfer *RNA* (*tRNA*) genes, the green is protein-coding genes (PCGs), and the orange is ribosomal *RNA* (*rRNA*) genes.

After assembly, the complete mitogenome of *O. colon* has been found to be 16,544 bp in length, which can be assembled into one circular contig (Figure 2). The mitogenome of *O. colon* is similar to other nitidulid beetles in terms of gene composition (Wu et al. 2019), containing 22 *tRNA* genes, 13 PCGs, two *rRNA* genes, and a non-coding region (Table 2). In addition, they also shared parallel gene arrangement: eight *tRNA* genes (trnC, F, H, L2, P, Q, V, and Y), four PCGs (ND1, ND4, ND4L, and ND5), and two *rRNA* genes (*l-rRNA* and *s-rRNA*) were located in the light strand, while the other genes were located in the heavy strand.

**Table 2.** Organization of the *O. colon* mitogenome.

Gene	Position	Length (bp)	Intergenic sequence (bp)	Start codon	Stop codon	Strand
*trnI(gau)*	1233–1296	64	−3	–	–	+
*trnQ(uug)*	1294–1362	69	−1	–	–	−
*trnM(cau)*	1362–1430	69	−15	–	–	+
*ND2*	1416–2435	1020	−2	ATC	TAA	+
*trnW(uca)*	2434–2498	65	5	–	–	+
*trnC(gca)*	2504–2565	62	4	–	–	−
*trnY(gua)*	2570–2636	67	−8	–	–	−
*CO I*	2629–4173	1545	−5	ATT	TAA	+
*trnL1(uaa)*	4169–4233	65	0	–	–	+
*CO II*	4234–4921	688	0	ATT	T	+
*trnK(cuu)*	4922–4991	70	0	–	–	+
*trnD(guc)*	4992−5055	64	0	–	–	+
*ATP8*	5056−5211	156	−7	ATT	TAG	+
*ATP6*	5205−5879	675	−1	ATG	TAA	+
*CO III*	5879−6666	788	−1	ATG	TA	+
*trnG(ucc)*	6666−6730	65	0	–	–	+
*ND3*	6731−7084	354	6	ATT	TAA	+
*trnA(ugc)*	7091−7156	66	0	–	–	+
*trnR(ucg)*	7157−7221	65	−1	–	–	+
*trnN(guu)*	7221−7284	64	0	–	–	+
*trnS1(ucu)*	7285−7351	67	0	–	–	+
*trnE(uuc)*	7352−7416	65	0	–	–	+
*trnF(gaa)*	7417−7483	67	9	–	–	−
*ND5*	7493−9197	1705	0	ATT	T	−
*trnH(gug)*	9198−9261	64	−1	–	–	−
*ND4*	9261−10,588	1328	−7	ATG	TA	−
*ND4L*	10582−10,869	288	2	ATG	TAA	−
*trnT(ugu)*	10,872−10,936	65	0	–	–	+
*trnP(ugg)*	10,937−11,002	66	1	–	–	−
*ND6*	11,004−11,510	507	−1	ATT	TAA	+
*CYTB*	11,510−12,649	1140	–2	ATG	TAG	+
*trnS2(uga)*	12,648−12,715	68	17	–	–	+
*ND1*	12,733−13,683	951	1	ATT	TAG	−
*trnL2(uag)*	13,685−13,751	67	−38	–	–	−
*l-rRNA*	13,714−15,047	1334	–2	–	–	−
*trnV(uac)*	15,046−15,114	69	−2	–	–	−
*s-rRNA*	15,113−15,899	787	/	–	–	−

Intergenic sequence represents (+) values as intergenic nucleotides and (–) values as overlapping regions.

The total length of the intergenic sequences in *O. colon* is 43 bp, with the longest intergenic sequence located between *trnS2* (*uga*) gene and ND1 gene with 17 bp length. Furthermore, the total length of the overlapping regions in *O. colon* is 97 bp. All the overlapping sequences of the mitogenome of *O. colon* range from 1 to 38 bp, and the longest overlapping sequence is located between *trnL2* (*uag*) gene and *l-rRNA* gene with 38 bp. Compared to the mitogenomes of other nitidulid beetles, the total length of the intergenic sequences in *O. colon* seemed to be much shorter, and the total length of the overlapping sequences was similar (Wu et al. 2019).

The nucleotide composition of *O. colon* is A: 41.1%, T: 38.7%, G: 8.3%, and C: 11.9% (Table 3). Compared to the mitogenomes of other insect species, the nucleotide composition of *O. colon* is also biased on A and T composition (A + T: 79.8%). Furthermore, AT-skew of the mitogenome is slightly positive with a value of 0.029 while GC-skew is negative −0.178 for *O. colon*. In most of the metazoan mitogenomes, the strand skew biases are found to be weakly positive AT-skew and strongly negative GC-skew (Li et al. 2017), which is found in this study as well in case of *O. colon*.

**Table 3.** Nucleotide composition of *O. colon* mitogenome.

Feature	A + T (%)	AT-skew	G + C (%)	GC-skew
Mitogenome	79.8	0.029	20.2	–0.178
PCGs	78.0	−0.140	22.0	–0.010
*tRNA* genes	79.5	0.030	20.5	0.154
*rRNA* genes	82.0	−0.059	18.0	0.333

### 3.2. Protein coding genes and codon usage

The size of 13 PCGs is 11,193 bp in the *O. colon* mitogenome, with 33 bp for stop codons. The majority of PCGs are encoded by the H strand and only ND1, ND4, ND4L, and ND5 are encoded by the L strand as shown in Table 2. Three types of start codons – ATT, ATC, and ATG – are used, of which ATT is the most common used start codon, and only *ND2* gene starts with ATC. Additionally, there are four types of stop codon: TAA, TAG, TA, and T, of which TAA is the most commonly used, while *CO II*, *CO III*, *ND5*, and *ND4* genes stop with incomplete stop codon TA or T. Incomplete stop codons are common in animal mitochondrial DNA and are likely to be completed by post-transcriptional polyadenylation (Ojala et al. 1981).

**Figure 3.**
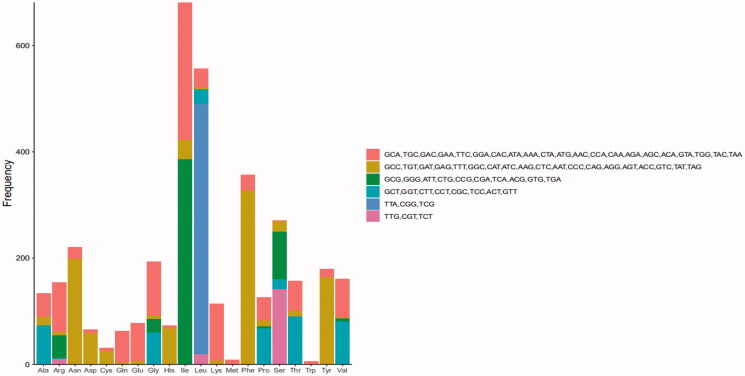
Relative synonymous codon usage (RSCU) for the *O. colon* mitogenome.

Figure 3 shows the RSCU of the *O. colon* mitogenome. Codon usage analysis indicated that the most frequently used codons in the *O. colon* mitogenome were TAA, ATT, TTT, ATA, and AAT. The frequent use of A and T in codon contributed to the high AT content in the *O. colon* mitogenome.

### 3.3. tRNA and rRNA genes

There are 22 *tRNA* genes in the mitogenome of *O. colon*, which are dispersed among the PCGs and *rRNA* genes. Among them, eight tRNAs lie on L strand, and 14 tRNAs lie on H strand. The positions and sizes (62–72 nucleotides) of tRNAs in the mitogenome of *O. colon* follows the typical organization for insect mtDNA.

**Figure 4.**
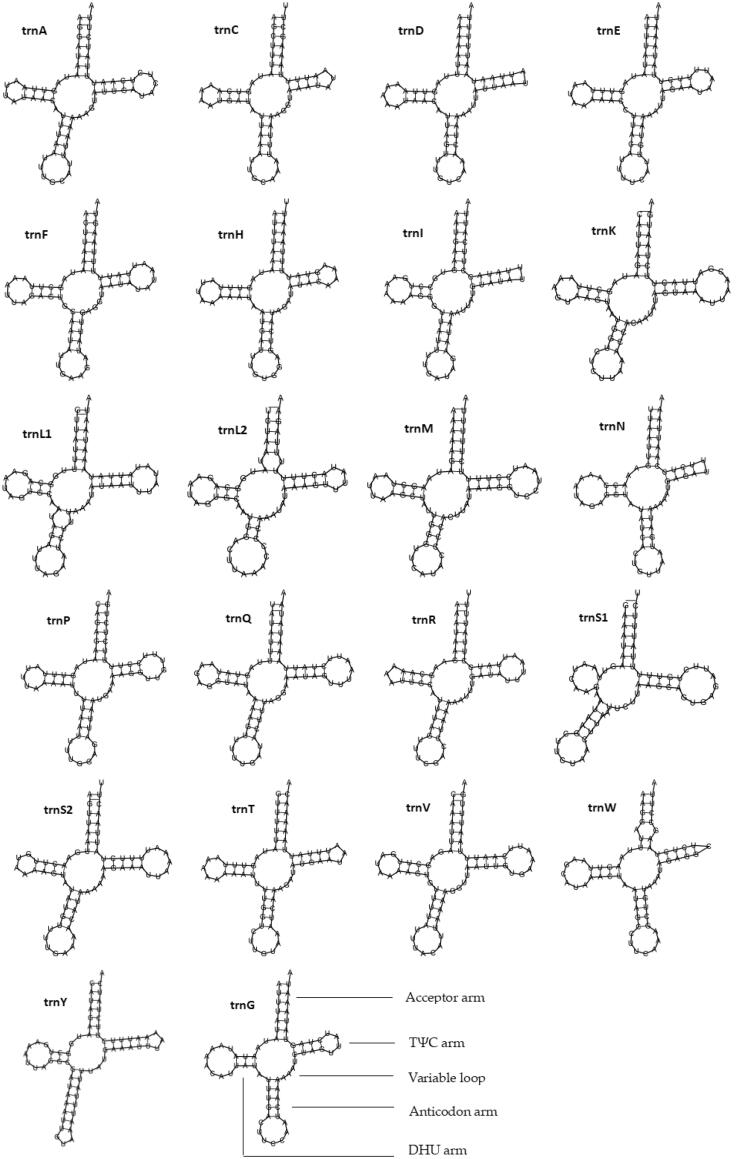
Putative secondary structures of tRNAs found in the mitogenome of *O. colon*.

Most tRNAs could be folded into the clover-leaf secondary structures including the aminoacyl (or acceptor) arm, dihydrouridine (DHU) arm, anticodon arm, and pseudouridine (TΨC) arm, except for trnD, trnI, trnN, and trnW, which lacked the TΨC-loop; meanwhile, trnS1 lacked the DHU arm (Figure 4).

The *l-rRNA* gene of *O. colon* mitogenome consists of 1334 nucleotides (position: 13714 − 15047), and the *s-rRNA* gene consists of 787 nucleotides (position: 15113–15899). In accordance with other insect mitogenomes, *rRNA* genes in the *O. colon* mitogenome are located in the L strand between the *trnL2* (*uag*) gene and the control region, separated by the *trnV* gene.

### 3.4. Phylogenetic analysis

**Figure 5.**
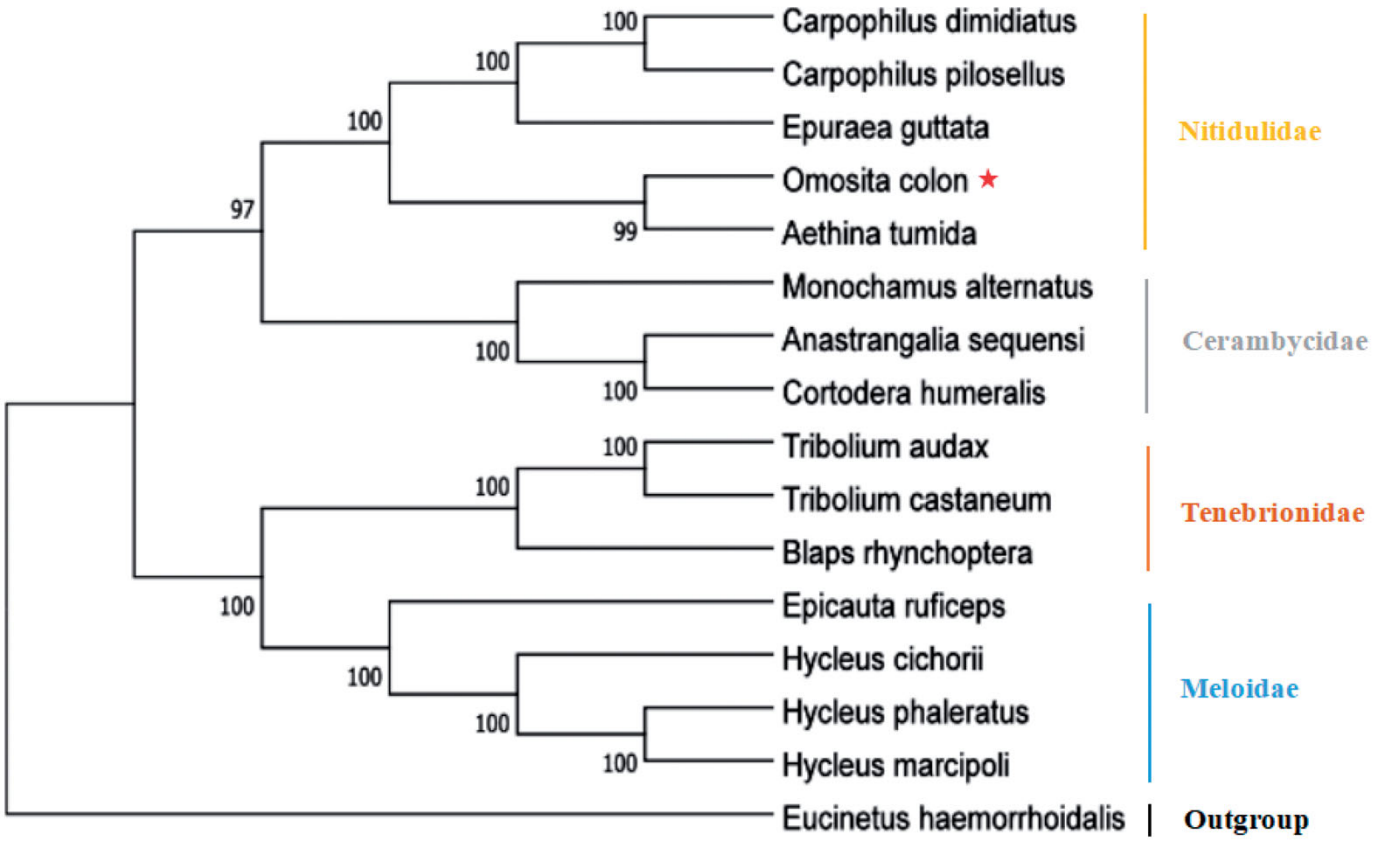
Molecular phylogenetic analysis of infraorder Cucujiformia by ML method based on complete mitogenomes.

The ML phylogenetic analysis with 15 complete mitogenomes (one generated in this study and 14 obtained from the GenBank) was conducted with MEGA version 10.0 (Figure 5). As shown in the figure, the clustering results of each branch were consistent with those of the taxonomy. Phylogenetic analysis results strongly supported that *O. colon* was closely related to *Aethina tumida*. The results indicate that *O. colon* and four other species of Nitidulidae form a clade, and that the monophyly of the family is well recovered.

## 4. Discussion

The complete mitogenome data is considered useful for genetic identification and phylogenetic studies. Furthermore, *O. colon* is an economically important storage pest worldwide and a forensically important beetle as well. In this study, we reported the first complete mitogenome sequencing of *O. colon*, and the result provided a molecular basis for species identification and inferring the PMI_min_ with *O. colon*.

We compared the mitogenomes of *O. colon* and other nitidulid beetles with respect to AT/GC contents, mitogenome organization, and codon usage patterns. We found that most of these features were similar, which meant that the gene order and other structural features of nitidulid beetles were largely conserved. In addition, we observed that the mitogenome of *O. colon* was very similar to other Coleoptera species in terms of gene organization, order, and size. For example, the length of *Dermestes tessellatocollis* (Coleoptera: Dermestidae) mitogenome is 16,218 bp, containing 13 PCGs, two *rRNA*s, and 22 *tRNA*s as well (Karagozlu et al. 2019). The similarities between these mitogenomes might be considered as common characteristics in case of Coleoptera species.

The arrangement of mitochondrial genes is an important reference for revealing the phylogenetic relationships among the species (Bo-Ying et al. 2018). The order of mitochondrial genes in animals has been extensively studied, and some models have been proposed to explain this rearrangement (Marleen et al. 2008). In this study, the first mitogenome sequence for species within the genus *Omosita* was reported, and more data from other species in this genus will be needed for carrying out further research. Mitogenome sequences will enable the resolution of species identification, phylogenetics studies, and molecular evolution of family *Nitidulidae* (Wu et al. 2019), which would supplement further researches in similar field.

## Data Availability

Mitogenome data supporting this study are openly available in GenBank at: https://www.ncbi.nlm.nih.gov/nuccore/MT749275. Associated BioProject, SRA, and BioSample accession numbers are https://www.ncbi.nlm.nih.gov/bioproject/PRJNA670574 https://www.ncbi.nlm.nih.gov/sra/SRR12879478 and SAMN16513378, respectively.
